# Uncooked fish consumption among those at risk of *Opisthorchis viverrini* infection in central Thailand

**DOI:** 10.1371/journal.pone.0211540

**Published:** 2019-01-31

**Authors:** Picha Suwannahitatorn, Joanne Webster, Steven Riley, Mathirut Mungthin, Christl A. Donnelly

**Affiliations:** 1 Department of Parasitology, Phramongkutklao College of Medicine, Bangkok, Thailand; 2 Centre for Emerging, Endemic and Exotic Diseases (CEEED), Department of Pathology and Pathogen Biology, Royal Veterinary College, University of London, London, United Kingdom; 3 MRC Centre for Global Infectious Disease Analysis, Department of Infectious Disease Epidemiology, Imperial College London, London, United Kingdom; 4 Department of Statistics, University of Oxford, Oxford, United Kingdom; Sciensano, BELGIUM

## Abstract

In contrast to northern and northeastern Thailand, central Thailand was believed not to be endemic for *Opisthorchis viverrini* (OV). Fieldwork conducted in a rural area of central Thailand revealed that the prevalence and incidence were relatively high compared with regional average data. We hypothesized that the behavioural-psycho-social background of the study population might play an important role in the high burden of the infection. As a result, a qualitative study was conducted to highlight potential social determinants of the infection dynamics to gain greater understanding of the risk behaviours and their contexts. A qualitative study using focus group discussion and in-depth interviews was conducted in Na-ngam Village, Chachoengsao Province from 2012–14. Framework analysis was used to explore associations between infection and thematic content. Social influence showed a strong impact on infection dynamics of OV infection. Our results revealed that *Koi pla* (chopped raw fish salad) remains a popular dish in the community, as the dish itself represents northeastern culture. The cultural norm had been transferred from ancestors to their descendants. Some elders complained that discontinuing the consumption of *Koi pla* went against old traditions with respect to cultural norms and socialization. In contrast, modern education teaches about hygiene including OV infection risks, and accordingly teenagers and young adults were reported to modify their lifestyles including their eating habits. Children are a potential key to pass knowledge to their parents and school-based education programs can serve as a practical hub for knowledge dissemination. However, health education alone might not lead to behavioural change in other age groups. Therefore, more efforts are needed to support the transformation.

## Introduction

*Opisthorchis viverrini* (OV), a human liver fluke, is a pathologically and economically important food-borne trematode. [1–3] OV infection is endemic in Southeast Asia along the Mekong Basin [4–7] including Thailand, Lao PDR and Cambodia, where an estimated nine million people are infected [8–10]. Transmission to humans (and other animals) occurs through the consumption of uncooked cyprinoid or white-scale freshwater fish containing infective stage metacercariae [11,12]. After infection, OV survives in the bile duct in the absence of treatment [13–15]. Many studies have demonstrated that chronic infection is strongly related to a bile duct cancer, cholangiocarcinoma (CCA) [16–19]. The International Agency for Research on Cancer has declared that OV is a group 1 agent, carcinogenic to humans [8]. Thailand has the highest CCA incidence in the world, with estimates ranging from 93.8 to 317.6 /100,000 person-years [13,18,20–22]. However, OV infection is acknowledged as a neglected and underestimated disease globally [9,10,23].

In Thailand, endemic areas are confined to locations where intermediate hosts are present and uncooked fish dishes are consumed [24,25]. The prevalence is high in north and northeast Thailand where eating habits closely follow local traditions. Incorporated in the National Public Health Development Plan from 1987 to 2001, the National Control Strategy was designed and implemented following three main approaches. [1,26] First, mobile teams provide stool examination and treatment in communities. Second, heath education promotes cooked fish consumption and hygienic defecation and last, community members prepare and participate in health campaigns.

The annual mobile stool examination strategy is used to prioritize case seeking and treatment. When the estimated prevalence has decreased below 10% in any community, the program is switched to a passive surveillance strategy National prevalence was estimated to be 63.6% at the start of the 6^th^ National Public Health Development Plan in 1991, [1]and the program succeeded in reducing prevalence. From 1997, the prevalence fell below 10% and remained below 10% for five consecutive years through 2001. Therefore, the program was considered to have achieved its goal and then switched to a passive surveillance strategy. From the 9^th^ National Public Health Development Plan to the current 11^th^ plan (2001 up to the present), the control strategy has been merged with rural health services, focusing on targeted areas where the infection burden remains high. Therefore, the disease control priorities vary by province. Knowledge, attitudes and perceptions regarding liver fluke infections have been evaluated in rural areas of northeastern Thai and neighboring Lao provinces. [25,27] The results showed that improving health education together with treatment could result in better knowledge and behavioral change concerning liver fluke infections[27]. The Health Belief Model (HBM) was introduced as a background theory to design a evidence-based systematic program.[28] The Behavior Modifying Program for Liver Fluke and CCA Prevention using HBM and social support was introduced in 1997 by Glanz et al.[29] The program included improving health education and initiating group activities to achieve behavioral modification and self-efficacy. The following Lawa Project using an integrative EcoHealth/One Health approach was later implemented in the northeast as a model using multidisciplinary approaches[30]. Knowledge, attitudes and perceptions played an important role in the behavioral-psycho-social compartment in these programs. However, the implementation of the program is still limited to the study area located in a liver fluke endemic area in northeastern Thailand. Incidence of OV infection was estimated to be 21.6/100 person-years from 2002 to 2004[31] and 21.4/100 person-years from 2007 to 2009[32] in Tha-kradan Subdistrict, Sanamchaikhet District, Chachoengsao Province, Central Thailand. The local population is a northeastern-originated community where local traditions are well-preserved. From questionnaire assessment, consumption of *Koi pla* (a popular northeastern dish in which freshwater fish is instantly chopped and mixed with spicy ingredients) was determined to present a potential risk factor for acquiring OV infection [12,31,32].

We hypothesized that the behavioral-psycho-social background of the study population played an important role in the high burden of OV infection. Therefore, we aimed to evaluate the ongoing risk behaviors, even in currently purportedly non endemic areas.

## Materials and methods

The research protocol of this study was approved by the Ethics Committee of the Royal Thai Army Medical Department (approval code S045h/54). Study participants or parents of participants under 18 years of age agreed to join the study after reading the data sheet provided in Thai and provided their written informed consent.

Study participants were enrolled from fieldwork surveys. A cross-sectional study was conducted in Na-ngam Village to estimate the baseline prevalence of OV infection in 2012. In 2014, follow-up incidence was estimated from a cohort study of participants who tested negative for OV infection at baseline.

### Collecting qualitative data

Research participants were invited to join focus group discussions (FGD). [33] The selection method was purposive sampling based on basic demographic data including sex, age and occupation and risk behaviors for acquiring the infection obtained from fieldwork surveys.

The enrolled participants were categorized in five groups as shown in Table 1 based on stool examination results from the 2012 to 14 survey study and health volunteer status. For the re-infected group, infected cases from the baseline study were treated with praziquantel (40 mg/kg/day, single dose) and assumed to be cured as posttreatment stool examination was not conducted. When their follow-up stool examination results were positive, they were assumed to be re-infected during the follow-up period. The local health volunteers who acted as fieldwork collaborators were assigned to separate groups to avoid them dominating the discussions. Members making interesting contributions to the group discussions were invited for in-depth interviews to provide more details. The FGD moderator used open-ended questions focusing on various aspects relating to OV infection and its risk factors. The flow of FGD was carried out until no additional contexts observed following these main themes in Table 2.

**Table 1:** Categorization for FGD participants based on stool examination results for OV infection and health volunteer status.

	**1**	**2**	**3**	**4**	**5**
	**Never infected**	**Previously infected**	**Newly infected**	**Re-infected**	Local heath volunteers
**Baseline study 2012**	Negative	Positive	Negative	Positive	
**Follow-up study 2014**	Negative	Negative	Positive	Positive	

**Table 2:** Main themes for focus group discussion.

**Knowledge**	• Basic knowledge of the infection; liver fluke life cycle, mode of transmission and infection, risk factors, diagnosis and treatment • Health-related consequences of the infection; role of carcinogens, cholangiocarcinoma
**Attitude**	• Perceptions towards OV infection and its consequences • Health concerns about the risk factors and the infection
**Uncooked fish consumption behaviors**	• Uncooked fish consumption in the community in all forms such as kind of fish, method of cooking and preservation • Current patterns of consumption behaviors regarding social aspects of the community
**Impact of the infection on the community**	• Health and social impacts from individual to community levels
**Management**	• Roles of the National Control Strategy from the community perspective • Concerns regarding treatment and control • Roles of primary prevention • Accessibility to healthcare services

### Data processing and analysis

The conversations were recorded using a smartphone. Data processing and analysis included multiple approaches to interpret the content of the conversations and exploring and explaining the situation of OV infection in qualitative aspects. The methods used included direct quotations and selected words to evaluate the actual local discourse used by the participants. The conversations were recorded in audio files and transferred to computer. Conversation from each session was manually transcribed to text using a word processing program. Then text-based data were sorted among predefined main themes[27,29,34,35]; knowledge, attitudes, uncooked fish consumption behaviors, impact on the community and solutions. In addition, relevant themes emerging from the analysis were also considered. The analysis included exploring interactions between factors regarding categorized thematic contents.

## Results

A total of 35 study participants were enrolled. FGD sessions were categorized in four groups; local health volunteers, never infected, newly infected and previously infected groups (Table 3). Participants in the re-infected group were too few to form a group discussion, so they were allocated to in-depth interviews to provide information about the recurrence of the disease.

**Table 3:** Characteristics of FGD participants.

Characteristic	Discussion group			
	Local health volunteer	Never infected	Newly infected	Previously infected
**No. of members**	12	9	7	7
**Sex**				
**Male** **Female**	1 (8.3%) 11 (91.7%)	1 (11.1%) 8 (88.9%)	5 (71.4%) 2 (28.6%)	1 (14.3%) 6 (85.7%)
**Mean age (years)**	39.8	49.8	62.8	56.0
**Age range (years)**	36–47	18–64	7–70	48–72
**Occupation**	Agricultural work	Agricultural work Self-employed Student	Agricultural work Self-employed	Agricultural work Self-employed
**Educational level**	Primary school	Primary school	Primary school	Primary school

From a total of 35 participants; 8 were male (22.9%) and 27 were female (77.1%). Overall mean age of participants was 51.1 years with a range of 7–70 years. Most were agriculture-related workers. The most common educational achievement level was primary school.

For in-depth interviews, 5 participants from 3 categories were enrolled, i.e., re-infected (n = 2), previously infected (n = 2) and newly infected groups (n = 1) with an age range of 7–83 years. The major occupation was self-employed. The main educational level was primary school.

Details from FGD and in-depth interviews are summarized following main themes.

### Knowledge of the infection

Participants knew that uncooked Cyprinoid fish contained metacercariae, which could lead to infection. However, some confused the life cycle with that of other parasites, and they perceived that the mode of infection was through the fecal-oral route, the common intestinal helminth life cycle. Teenagers demonstrated better knowledge of OV infection. They knew the important parts of the life cycle and could explain the mode of infection and transmission. However, most of their knowledge was acquired from textbooks or the internet. They stated that they only knew of the parasite without experiencing it which might have affected their awareness. The adult and elderly groups recognized that OV infection could lead to bile duct cancer, but some believed it was a result of agricultural insecticide contamination of fish meat or immunocompromised hosts as evidenced below.

“*I don’t see any association between the infection and cancer*. *In fact*, *chemical toxins have affects*. *I see many people who never smoke*, *drink or eat raw fish but still die of cancer*. *I think it depends on everyone*. *Of course*, *these materials have toxins*, *but if you were strong enough*, *you’ll be fine*. *It doesn’t matter what you take into your body*. *If your immunity was weak*, *you’ll get cancer even [if] you eat vegetables*,” One 70-year-old-man discussed the cause of cancer. A 62-year-old man from the newly-infected group also reported that, “*Some people have a very healthy lifestyle*. *Sadly*, *they died at 40 or 50 years from cancer*. *It doesn’t make any sense and it’s proven that they’re related*.”

### Attitude and perception of OV infection

The villagers perceived that OV infection was a major problem for the community. They showed informed attitudes about OV infection. Although some might have misunderstood about the life cycle, they agreed that cooked food was more hygienic and could prevent not only OV infection but other intestinal parasites as well. Their consumption behaviors might be difficult to observe in practice, but parents were trying not to let their children consume *Koi pla*. After receiving stool examination results, some participants decreased their uncooked fish consumption. Even when the result was negative, some were still concerned about others’ results or they were afraid that if they chose to continue risk behaviors, one day the result might be positive. As the villagers perceived that OV infection was a disease, they preferred to focus on treatment rather than prevention. In addition, some learned that treatment for the infection was simple and feasible so they were willing to wait for healthcare worker as they thought it should be the physician’s responsibility to take care of them.

### Uncooked fish consumption behaviors

Uncooked fish preparations[25] were classified in two major groups. The first constituted instantly prepared uncooked fish, a well-known popular dish known as *Koi pla* (chopped raw fish salad). This freshwater fish is chopped and mixed with spicy ingredients. Raw fresh meat is usually denatured by the acidity of lime juice, which dramatically changes the color and texture of the fish. *Koi pla* is always consumed instantly. The second dish was extensively fermented fish, known as *Pla ra*, a freshwater fish preserved in a highly-concentrated salt solution for 3 to 6 months which metacercariae could not survive [32]. Chunks of fish could be consumed uncooked and the remaining fish sauce was often used as daily ingredient for local dishes. Most villagers knew that some of their popular fish dishes were uncooked. Some participants believed that all uncooked dished could cause OV infection while others knew that only fresh fish material could lead to infection. They perceived that the infection was strongly associated with consumption behaviors and chronic infection could result in serious outcomes. However, misunderstandings and misconceptions remained among some villagers.

### Impact of the infection on the community

Older adults showed strong attachment to the traditional northeastern values and culture. Adult participants acknowledged that instantly-prepared uncooked fish could cause infection. Younger generations loved to eat instantly prepared foods such as instant noodles or ready-to-eat meals. Some thought it was fashionable to eat what was advertised on the television. Some knew that *Koi pla* dish was a risk factor, so they avoided eating *Koi pla* but sought a substitute dish. Some believed that all kinds of uncooked fish dishes led to infection, so they chose to continue their eating behaviors unchanged because modifying a familiar lifestyle was too complicated.

*Koi pla* was shown to be strongly related to multiple aspects of their lifestyle representing traditional values. It was a food frequently eaten with alcoholic drinks especially in male social drinking. Some stated that eating *Koi pla* was related to agriculture-related occupations. Some families in which parents still consumed *Koi pla* kept their children away from it unless cooked. Moreover, when children and teenagers consumed *Koi pla*, they were always from families with uncooked fish-eating habits. In contrast, *Pla ra* consumption was popular at all ages and both sexes. *Pla ra* was used as a seasoning, food ingredient or even consumed as a main dish. The roles of *Pla ra* are various. When mentioning uncooked fish dishes, *Pla ra* would be the very first dish they recognized.

Health education provided more insights about the threat of uncooked fish consumption. The roles of local health volunteers and health campaigns influenced the behavioural patterns of consumption. High incidence of OV infection triggered concerns about the public health consequences for the community. However, participants suggested that the infection was typically asymptomatic and the symptoms that did occur were nonspecific. Because infected individuals were physically healthy, they might not have been aware of infection.

All groups expressed that they were asked for stool examination because the doctors (research team) thought it was important. Nobody ever asked for a stool examination at the local healthcare facility. Teenagers and adults knew that chronic infection could lead to cancer, but they did not know exactly what kind of cancer resulted from OV infection. Moreover, the name “liver fluke” (also called in Thai) confused the villagers and that the pathology occurred in liver tissue. Some believed that consuming *Koi pla* with alcohol could accelerate the process of liver cirrhosis. Elderly members thought OV infection was unrelated to cholangiocarcinoma. They observed that many were still healthy even though they regularly consumed uncooked fish. From discussions, they suggested uncooked fish consumption needed to be slowly reduced; as abruptly discontinuing consumption seemed impractical.

### Management

When villagers were aware of being infected, two approaches allowed them to receive medication. Firstly, they waited for the stool examination result from the research team who regularly visited them. Secondly, they also perceived that anthelminthic medication was available at the community healthcare centre or they could directly purchase over-the-counter medicine from the pharmacy at their convenience. Unfortunately, those anthelminthic drugs, such as albendazole or mebendazole, were for intestinal helminths. Praziquantel was not available in the community pharmacies unless villagers went to the district hospital which was one hour’s travel away. However, some who took medicine still recognized praziquantel from its side effects. Moreover, some stated that a home remedy was available to cure parasitic infection.

The National Control Strategy has focused on reducing consumption of all uncooked fish dishes to interrupt the infection process; therefore, it could potentially prevent the occurrence of cholangiocarcinoma from OV infection. However, consumption behaviors were strongly attached to the local culture. Children had a better understanding about the knowledge of infection. Some adults and elderly felt more comfortable and hesitated less when educated from their offspring. Some of participants observed that children could serve as an effective medium for transferring knowledge. Additionally, they did not want their children to practice the same habit. Most villagers had toilets in their house. Some installed toilets on their farm. Otherwise, they would defecate in the field and cover with soil. Just a few people defecated directly in the natural water resource.

### Model framework

The main findings from the predefined framework could be approached with age structure as children, teenagers, adults and the elderly where we have observed that the process of knowledge and culture transfer showed some interesting characteristics.

**Fig 1:**
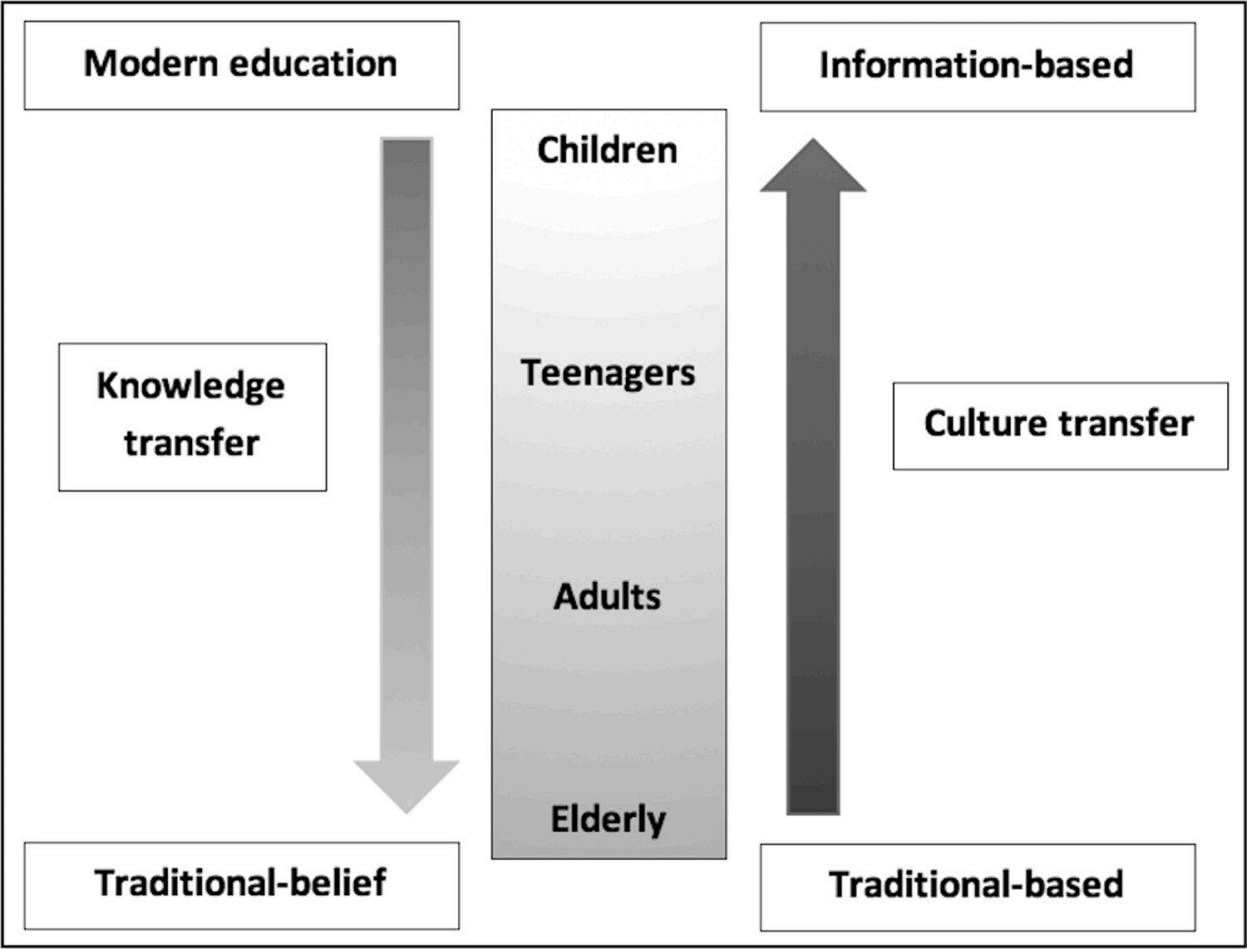
Model framework for knowledge and culture transfer.

A model framework is proposed in Fig 1, illustrating how social components could play a major role in influencing the infection dynamics regarding behavioral-psycho-social aspects. Regarding the overall picture, basic knowledge of OV infection required modern education usually provided by formal resources such as schools. Some traditional beliefs still played important roles concerning knowledge of infection. Knowledge and attitudes about OV infection affected risk behaviors and perceptions concerning prevention and control. The pattern of knowledge transfer was likely to start with children and teenagers when their parents agreed to let them have modern education from school. Younger generations could access more informative sources such as the internet; and therefore, could pass modern knowledge to the older generation, i.e., their parents and the elderly.

## Discussions

The study provided more insight about OV infection dynamics emphasized on uncooked fish consumption behaviors in a behavioral-psycho-social aspect. However, assumption was mainly made by data obtained from FGD in 35 participants with majority of female participants (77.1%), and in-depth interviews in 5 participants. Therefore, strong conclusion should be aware to reflect the whole picture of rural Thai community, and would be discussed in relation to the data obtained from this study. Additionally, microscopic-based stool examinations could not differentiate eggs of O. *viverrini* from those of minute intestinal flukes in terms of morphological appearance. The issue has been described in previous cohort study in Na-yao area, the adjacent community, from 2007 to 09 [32] and molecular study [36] showed that minute intestinal flukes were not detected by PCR.

In this community, the local northeastern culture still exhibits a strong influence on attitudes and perceptions about risk behaviors passed on from previous generations. This complex relationship showed that younger generations with modern education tend to have multiple sources of information and started justifying the risks and benefits from practicing risk behaviors. The interaction between modern information and traditional beliefs could be observed during transitional periods among teenagers and early adults when self-esteem and independence start to develop as shown in Fig 1. Importantly, villagers could understand that the mode of infection was consumption of uncooked fish. The consumption of instantly prepared dishes such as *Koi pla* constituted a risk factor. The parasites live in the body for many years, son infected individual may not be aware of the asymptomatic, chronic infection leading to cholangiocarcinoma. The risk of infection is higher when practicing risk behaviors, so avoiding uncooked fish consumption was the preferred primary prevention.

It was reported that school played a major role as an information hub equipped with academic resources such as teachers, books and internet access. Therefore, most health knowledge among young children and teenagers was likely school-based. Teenager behaviors tend to be motivated by social influence; they are naturally sensitive to information with respect to physical and psychological development during puberty. Therefore, they are more likely to follow social trends including eating habits. From the study, the modern lifestyle leads them to choose more urbanized food. However, teenagers choose more independently. In terms of eating habits, they might choose to consume uncooked fish when they perceived that their previous generation was a role-model. From their information-seeking behaviors, they might have to reconcile the knowledge they had about OV infection and how they imitated others or developed life-long eating habits. Teenagers and adults also required evidence. For instance, they asked the research team if they could be shown actual liver flukes or eggs. Data presentation from health professionals also focused their attention on the infection.

The elderly had barriers to learning new knowledge, especially when new knowledge conflicted with their traditional beliefs. This issue also occurred among adults as they did not pay much attention to the information provided from youth. However, data analysis showed that age barriers could be minimized. Knowledge transfer could be performed more smoothly by family members, and adults showed less hesitation when the information was provided by their offspring. This connection provided the mechanism for the transfer of school-based knowledge to the community by school children as a medium creating a practical strategy for knowledge distribution.

An earlier quantitative study in Na-yao area [32] indicated that the National Control Strategy may have had some issues regarding promoting the prevention of uncooked fish consumption campaign [37,38]. The participants found that avoiding all uncooked fish dishes was impractical as indicated by the guidelines provided, so they were more likely to continue the same eating habits resulting in continued *Pla ra* consumption. *Pla ra* was widely used as a main ingredient for local dishes, so the indirect impact from unspecified uncooked fish avoidance campaign resulted in continued *Koi pla* consumption.

A relationship was established between states of dependence and independence referring to adulthood and childhood; while they contributed their own decisions. Most forbade their children from consuming uncooked fish. In some cases, all family members consumed *Koi pla* on a regular basis. Adults were reported to show more concern about physical function than general appearance. They had to maintain physical fitness to perform work almost every day. Moreover, some risk behaviors were considered rewards after a long and hard day at work such as social drinking or consuming *Koi pla* considered a tasty dish along with alcohol. Many chronic diseases begin during adulthood. Some conditions such as hypertension and diabetes are asymptomatic at the early phase. Study results suggest that OV infection shares this feature and likely affects public health awareness. Adults were more likely to be concerned about what would immediately compromise their fitness such as myalgia or trauma. The elderly thought that healthcare should be under a doctors’ supervision. Their ideas represented a passive defensive strategy where they only sought healthcare after contracting illness. They also justified the efforts compared with the benefits for behavioral modification. The benefits from primary prevention may not be as visible because the disease is prevented rather than when an illness is cured. However, the elderly also perceived that taking praziquantel as a treatment for OV infection caused more illness than being infected and remaining asymptomatic. Nausea and dizziness were unfavorable outcomes.

In some cases, they thought that healthcare should only involve drugs, medical equipment and healthcare workers. As a result, primary prevention was not regarded as a part of healthcare; and because prevention was less prioritized, treatment became increasingly important. Lack of motivation was an important issue for both prevention and treatment. Moreover, even when they understood fatalities resulted from cholangiocarcinoma, it could be a result from chronic infection occurring many years ago. Considering their remaining years of age, the elderly calculated having cancer now was unlikely.

## Conclusions

Social influence plays an important role in shaping the dynamics of OV infection. The interaction of knowledge and culture transfer across generations provided insights into behavioral-psycho-social dynamics in affected communities. *Koi pla* was considered a tasty dish, which many people considered an important factor in its continued consumption. To some the dish itself represents northeastern culture. The elderly generation recently migrated from northeastern Thailand, so traditional beliefs were transferred from their ancestors to their descendants. They still preserved their traditional lifestyle even though they had migrated. However, the younger generation was more likely to absorb modern culture and become more urbanized. In rural settings, community leaders are important keys in the social structure. As the local administration is managed by the central and regional authority, governmental staff might not be aware of local cultural norms. Community leaders could bridge this gap and help facilitate the government’s strategies.

Medical doctors and healthcare personnel are also considered to be community leaders looking after villagers’ health. People respect and trust doctors when they are instructed and advised regarding their health. However, at present, doctors are not permanently stationed at some rural health centers. They visit the community once weekly mostly regarding chronic disease appointments. In Thailand, the shortage of healthcare workers is still a major problem in rural areas. Local health volunteers played important roles facilitating communication between villagers and health authorities. They could act as community leaders and role models for health interventions. Because they are local, they could more easily gain trust and cooperation from the villagers, a crucial part of the sustainability of health campaigns in the community [27,35]. Health education alone has been insufficient to motivate the behavioral changes required throughout the population. More efforts are needed to support this transformation. Children represent a potential key to pass knowledge to their parents and schools are practical hubs for knowledge distribution.

## Supporting information

S1 File Interview guide. (PDF)

S2 File Study’s minimal underlying data set. Thematic contents.
(PDF)
