# Supplementary material for: Uncooked fish consumption among those at risk of Opisthorchis viverrini infection in central Thailand
Source: PLoS One. 2019 Jan 31;14(1):e0211540. doi: 10.1371/journal.pone.0211540 (PMC6355008; doi:10.1371/journal.pone.0211540)
Supplement: S1 File — (PDF) [file pone.0211540.s001.pdf]

Supporting information - Interview guideline

Focus group discussion

Research participants were invited to a group conference or interview in a focus group discussion. The selection method was stratified purposive sampling. The enrolled participants were categorized in 5 groups based on stool examination results for OV infection. Demographic characteristics were considered the selection criteria based on homogeneity of the study population to avoid influential members leading or dominating the conference. However, the enrolment process was flexible and preliminary interview was given to demonstrate attitudes and opinions of the invited members.

Table 1 Selection criteria for focus group discussion

	**1**	**2**	**3**	**4**	**5**
	**Never-infected**	**Previously-infected**	**Newly-infected**	**Re-infected**	
**Baseline study 2012**	Negative	Positive	Negative	Positive	Local heath volunteers
**Follow-up study 2014**	Negative	Negative	Positive	Positive	

As shown in Table 1, the main criteria were based on stool examination results showing infection dynamics during the follow-up period reflecting the corresponding factors contributing to the infection. For the re-infected group, infected cases from baseline study were treated with praziquantel and assumed to be cured. If their follow-up stool examination result was positive, they were assumed to be re-infected during the follow-up period.

The local health volunteers who acted as fieldwork collaborators were categorized in separate groups to avoid them dominating the discussions.

For some unique cases, in-depth interviews were arranged for a specific case with interesting issues.

1. The stool examination result was analysed for classification criteria with respect to study results in 2012 and 2014.

2. The method of selection was purposive sampling. Joining the study was voluntary and written informed consent was given by the enrolled participants. The consent also included the permission for voice and image recording during the conversation.

3. Each participant was informed about their group, time and venue for the group discussion. They were notified again shortly before the appointment by any means of communication such as mobile phone or personal reminder.

4. On the conference day, an ice-breaking exercise was performed at the beginning of the meeting. The moderator would introduce him/herself and let each of the participants speak as an introduction to get to know each other and establish a friendly environment. Then participants were informed about the objectives and the process of the discussion.

5. The moderator managed the conversation by asking focused questions to meet the study objectives and maintain the flow of opinions expressed. The moderator maintained the structure of the conference while continuing the session in a relaxed atmosphere; the participants received equal opportunity to freely express their opinions.

6. Discussions focused on various aspects relating to OV infection and its risk factors. The flow of discussion followed the main topic themes listed below.

   - Knowledge
     - Basic knowledge of the infection; liver fluke life cycle, mode of transmission and infection, risk factors, diagnosis and treatment
     - Health-related consequences from the infection; role of carcinogens, cholangiocarcinoma
   - Attitude
     - Perception of OV infection and its consequences

    - Health concerns of the risk factors and the infection
- Uncooked fish consumption behaviours
    - Situation of uncooked fish consumption in the community
    - Current pattern of consumption behaviours regarding social aspects of the community
- Impact of the infection on the community
    - Health and social impacts from the individual to community levels
- Solutions
    - Roles of the National Control Program from the community perspective
    - Concerns regarding treatment and control
    - Roles of primary prevention
    - Accessibility to healthcare services

7. The moderator used open-end questions to contribute various expressions from the members. Closed-end questions were also used to clarify some interesting points.
8. The conversations were recorded using a voice recorder. The data collection proceeded continuously until the discussions were saturated when nothing new was being generated from the meeting. It usually took 30 – 45 minutes for each session.

In-depth interview

Some members were invited to face-to-face interviews. The conversations focused on specific points of interest raised during the focus group discussions.

แนวทางดำเนินการสนทนากลุ่ม

ผู้เข้าร่วมวิจัย จะได้รับเชิญให้เข้าร่วมการสนทนากลุ่มแบบเจาะจง (Focus group discussion) โดยผู้วิจัยจะแบ่งกลุ่มตัวอย่างให้คนในกลุ่มมีความคล้ายคลึงกัน (homogenous) เพื่อไม่ให้เกิดความมีอิทธิพลของคนใดคนหนึ่งในกลุ่ม กลุ่มสนทนามีประมาณ 10 - 12 คน และไม่ควรเกิน 15 คนเพราะคนจะมากเกินไป ทำให้การอภิปรายไม่ทั่วถึงเท่าที่ควร และถ้าน้อยกว่านี้จะทำให้เกิดการชี้นำได้ และยังทำให้ได้ข้อมูลที่จำกัด และไม่ครอบคลุมพอ ลักษณะของกลุ่มประชากรที่ทำการสนทนาแบบเจาะจงแบ่งได้เป็น 5 แบบ โดยอิงจากผลการตรวจอุจจาระเมื่อปี 2012 และการตรวจติดตามในปี 2014 ดังนี้

1. ผู้ที่ติดเชื้อใหม่ คือผู้ที่ไม่ติดเชื้อในปี 2012 แต่ตรวจพบการติดเชื้อในปี 2014
2. ผู้ที่ติดเชื้อซ้ำ คือผู้ที่พบการติดเชื้อทั้งในปี 2012 และ 2014
3. ผู้ที่ติดเคยติดเชื้อแต่หายแล้ว คือผู้พบการติดเชื้อในปี 2012 แต่ไม่พบการติดเชื้อในปี 2014
4. ผู้ที่ไม่เคยติดเชื้อเลย คือผู้ที่ไม่พบการติดเชื้อทั้งในปี 2012 และ 2014
5. กลุ่มอาสาสมัครสาธารณสุขประจำหมู่บ้าน

ผู้ดำเนินการสนทนา (Moderator) จะเป็นผู้คอยจุดประเด็นในการสนทนาเพื่อชักจูงให้กลุ่มเกิดแนวคิดและแสดงประเด็นหรือแนวทางการสนทนาอย่างกว้างขวางละเอียดลึกซึ้ง โดยมีผู้เข้าร่วมการสนทนาในแต่ละกลุ่มประมาณ 8 - 12 คน ซึ่งมาจากประชากรเป้าหมายที่กำหนดไว้ ซึ่งมีขั้นตอนดังนี้คือ

1. ให้ผู้เข้าร่วมเซ็นใบยินยอมการเข้าร่วมวิจัย

2. แจ้งข้อมูลแก่ผู้เข้าร่วมงานวิจัยในเรื่องของวัตถุประสงค์ของการเข้าร่วมในการสนทนากลุ่ม โดย “การเข้าร่วมกลุ่มสนทนาเพื่อให้แสดงความคิดเห็นและประสบการณ์เกี่ยวกับการรับประทานปลาดิบและพยาธิใบไม้ตับ และขอให้ทุกคนออกความเห็นอย่างเต็มที่ ขอให้ทุกคนพูดตามความคิดของตน ไม่มีถูก ไม่มีผิด”

3. การทำกลุ่มสนทนา โดยจะให้แต่ละคนภายในกลุ่มนั่งเป็นวงกลมและมีป้ายชื่อและหมายเลขติดไว้ โดยทำผังที่นั่งพร้อมชื่อของทุกคน โดยใน 1 กลุ่มสนทนาจะมีผู้ดำเนินการสนทนาและผู้บันทึกตำแหน่งละ 1 คน และจะมีอีก 1 คนคอยจัดการความเรียบร้อยในการสนทนาเช่น น้ำดื่ม อาหารว่าง และคอยให้คำแนะนำโดยสุภาพกับคนที่ไม่ได้รับเชิญเพื่อไม่ให้เข้ามาแทรกในวงสนทนา ใช้เครื่องบันทึกเสียงที่จะใช้ในการสนทนา จะมีผู้ดำเนินการสนทนาในการนำกลุ่มสนทนา สำหรับโครงสร้างของข้อคำถามนั้นมีจะมีรายการประเด็นสนทนาเป็นคำถามปลายเปิด และเตรียมคำถามปลายปิดไว้คร่าวๆ เพื่อใช้ในการเจาะประเด็น

4. การทำกลุ่มสนทนาจะใช้เวลาประมาณ 30 - 45 นาที โดยคำถามจะแบ่งเป็น 2 ส่วนคือข้อคำถามเกี่ยวกับบริบทของสังคมและข้อคำถามเกี่ยวกับโรคพยาธิใบไม้ตับ

    - ข้อคำถามเกี่ยวกับบริบทของสังคม จะถามจากผู้นำในชุมชน เจ้าหน้าที่อนามัย หรือผู้ที่มีอาวุโสของชุมชน โดยข้อคำถามจะถูกกำหนดเพื่อหาสิ่งเหล่านี้คือ พื้นเพดั้งเดิม การอพยพและสาเหตุของการอพยพ ประเพณี-วัฒนธรรมท้องถิ่น การประกอบอาชีพ เศรษฐฐาน และระบบสาธารณูปโภคและการอนามัยของชุมชน

- ข้อคำถามเกี่ยวกับโรคพยาธิใบไม้ตับ จะใช้ถามในการประชุมกลุ่มสนทนาแบบเจาะจงโดยข้อคำถามจะถูกกำหนดเพื่อหาสิ่งเหล่านี้คือ ความรู้เกี่ยวกับโรคพยาธิใบไม้ตับ ทัศนคติเกี่ยวกับโรค พฤติกรรมการบริโภคปลาน้ำจืดดิบ และแนวทางการแก้ปัญหา
- ผู้เข้าร่วมการสนทนากลุ่มที่มีประเด็นที่น่าสนใจ จะถูกเชิญให้เข้ารับการสัมภาษณ์เชิงลึกต่อไป
