# Supplementary material for: Uncooked fish consumption among those at risk of Opisthorchis viverrini infection in central Thailand
Source: PLoS One. 2019 Jan 31;14(1):e0211540. doi: 10.1371/journal.pone.0211540 (PMC6355008; doi:10.1371/journal.pone.0211540)
Supplement: S2 File — Thematic contents. (PDF) [file pone.0211540.s002.pdf]

Supporting information - Study's minimal underlying data set

Thematic contents

1. Situation of the OV infection in the community

Table 1 Situation of the OV infection in the community

Themes	Concerning topics	Emerging issues
• Acknowledgement of the infection<br>• Uncooked fish consumption was identified as health risk	• Cultural value and social influence of uncooked fish consumption<br>• Misunderstanding of uncooked fish menus	• Local health volunteers were regarded as respected members of community

Health campaign based on the National Control Program was provided to the community including health education, diagnosis and treatment by the collaboration between research team and local health volunteers.

Local health volunteers played an important role to communicate and interact among villagers and local health authorities. They gained respect from the villagers as important key members of the community.

As shown in Table 1, most participants acknowledged that OV infection was still a health burden to the community. They perceived that the infection was strongly associated with consumption behaviours and chronic infection could result in serious outcome. However, misunderstanding and misconception remained in some villagers. The potential of social and cultural influence also impacted on attitudes and perceptions of the infection. High-risk populations and risk factors continued to be identified. Moreover, the prevalence and incidence of OV infection were still relatively high compared with the baseline population.

Most villagers knew that some of their popular fish dishes were uncooked. Some were still confused that all uncooked dished could cause OV infection while some of them knew that only fresh fish material could lead to infection.

Koi pla (chopped raw fish salad) is a popular dish that has been consistently identified as a major risk factor of the infection. Although the community learned that it could lead to infection, the dish was still valued as a Northeastern culture norm. Daily consumption might have decreased; *Koi pla* consumption was still popular during festive events, friend or family meeting and male social drinking.

Older age showed strong attachment to the traditional value of Northeastern culture; "*Most of the elders still eat raw fish; they came from the Northeastern region*" said one 48-year-old woman from the newly-infected group mentioning the strong attachment between the elders and their ancestors who originally lived in Northeastern Thailand and later migrated to the current area.

2. Current situation of consumption behaviours

Table 2 Current situation of consumption behaviours

Themes	Concerning topics	Emerging issues
• Popularity of uncooked fish consumption<br>• Eating pattern among generation	• Subpopulations were identified for risk behaviours<br>• Burden in behavioural modification	• Some uncooked fish menus were the way of life<br>• Eating habit as family tradition

The current situation of consumption behaviours is shown in Table 2; adult participants acknowledged that instantly-prepared uncooked fish could cause the infection. Some of them knew that *Koi pla* dish was a risk factor, so they avoided eating *Koi pla* but sought a substitute dish. Some of them perceived that all kinds of uncooked fish dishes led to infection, so they chose to continue their eating behaviours unchanged because modifying a familiar lifestyle was too complicated.

Moreover, some dishes represented a traditional value, especially *Koi pla*, which they always consumed during festive events. Some elderly members stated that,

"*I've been living with this food for a long time; it's a part of my life. It's valuable. But children don't eat it anymore*"

"*When I have family meetings, if they serve you (Koi pla) you should eat it, or else you are insulting them*" said a 51-year-old woman from the newly-infected group.

Although uncooked fish dishes varied in this community, *Koi pla* and *Pla ra* were the most popular dishes villagers frequently consumed in both sexes and all age groups.

"*Everybody eats raw fish; it's very rare to see someone who doesn't*" said one member of the re-infected group. However, consumption of *Koi pla* decreased in females and at younger ages, "*As I recognize, it's once a year, twice as a maximum.*" said one 40-year-old woman describing having *Koi pla*.

Koi pla was shown to be strongly related to multiple aspects as their way of life. It was considered a food frequently eaten with alcoholic drinks especially in male social drinking. "*Koi pla was served in a drinking party among male friends. Koi pla was the best when paired with alcohol*", one 70-year-old man from the newly-infected group mentioned about the relationship between alcohol drinking and uncooked fish-eating habit. "*It's a socialization process,*" said one 61-year-old from the newly-infected group.

"*If I have to quit Koi pla, I have to quit alcohol, which is a highly unlikely thing for me to want to do*," said one 48-year-old man from the re-infected group.

Some of them stated that eating *Koi pla* was related to agriculture-related occupations: "*Farmers always eat Koi pla, fish is easily caught from the river when they go to a paddy field*" said one 40-year-old woman from the never-infected group.

On the contrary, *Pla ra* consumption was popular at all ages and both sexes. *Pla ra* was used as a seasoning, food ingredient or even consumed as a main dish. The roles of *Pla ra* are various. When mentioning uncooked fish dishes, *Pla ra* would be the very first dish they recognized. "*I can't live without Pla ra*," said one 48-year-old woman from the previously-infected group.

Some families whose parents still consumed *Koi pla* kept their children away from it unless cooked. Moreover, if children and teenagers consumed *Koi pla*, they were always from families with uncooked fish eating habits. "*I want to eat it*," one 7-year-old boy mentioned about *Koi pla*, who had parents who regularly consumed *Koi pla* and he was a newly-infected case.

Some villagers stated that uncooked fish consumption was an old tradition. It might be difficult to avoid or stop eating due to the strong cultural attachment. However, it began to gradually fade out from the younger generation as one 64-year-old women from the never-infected group said, "*They don't even recognize Pla ra; they just enjoy hotdogs.*"

The teenage group said that they did not like *Koi pla.* They thought the preparation method and food appearance looked disgusting and unacceptable. Moreover, the fishy taste did not match their more

urbanized lifestyle. Younger generations loved to eat instantly prepared foods such as instant noodles or ready-to-eat meals. Some thought it was fashionable to eat what was advertised on the television.

"*Teenagers do not have much uncooked food. They enjoy instant noodles to anything easy to prepare such as fried eggs*"

"*They like cooked food, whatever kind of preparation; fried, grilled or roaste*d"

Health education provided more insights about the threat of uncooked fish consumption. The roles of local health volunteers and health campaigns influenced the behavioural patterns of consumption.

"*The newer generation never eats raw fish; they were educated from their school*," said one 64-year-old woman from the never-infected group.

3. Knowledge of the infection

Table 3 Knowledge of the infection

**Themes**	**Concerning topics**	**Emerging issues**
• Causes of parasitic infection come from many routes<br>• Individual parasite needs its own mode of infection	• Inaccurate knowledge leads to risk behaviours<br>• Lack of visible evidence of parasite affect awareness	• Misinterpretation<br>• Information overload<br>• Healthy lifestyle does not guarantee healthy life

Table 3 shows that participants knew that uncooked Cyprinoid fish contained metacercariae, which could lead to infection. However, some of them were confused about the life cycle of other parasites;

"*When I go out barefooted, the worms penetrate my skin and go up to infect the liver*"

"*They go through your nail once you chop fish and leave the utensil unwashed*"

Some of them perceived that the mode of infection was through the faecal-oral route, the common intestinal helminth life cycle.

"*It's not about eating raw fish; it's about fresh vegetables. Don't you see eating raw fish was obviously reduced nowadays but people still become infected? I'm sure it was contaminated with vegetable*s," said one 51-year-old woman from the newly-infected group.

Some villagers perceived that fish from clean water sources was free of metacercariae.

"*The water body was clean without aquatic weeds or chemical substances, so the fish was also clean from parasites,*" said one 70-year-old from the newly-infected group.

They also knew that *Koi pla* was a risk factor for OV infection. Most of them understood that dressing uncooked fish meat with acidic agents such as lime juice or spirits only changed its colour, it was not completely cooked and metacercariae were still viable.

However, one member from the re-infected group had never heard about OV infection.

"*I knew nothing until the doctors came. It's very usual around here; fish are chopped and mixed with ingredients, tasty*," one 48-year-old man admitted.

Teenagers showed better knowledge of OV infection. They knew the important part of the life cycle and were able to explain the mode of infection and transmission. However, most of their knowledge was acquired from textbooks or internet. They stated that they only knew of the parasite without experiencing it which might have affected their awareness.

The adult and elder groups recognized that OV infection could lead to bile duct cancer, but some believed it was a result of agricultural insecticide contaminated with fish meat or immunocompromised host as evidenced below.

"*I don't see any association between the infection and cancer. In fact, chemical toxins do. I see many people who never smoke, drink or eat raw fish but still die of cancer. I think it depends on each individual. Of course, these stuffs have toxins, but if you were strong enough you'll be fine. It doesn't matter what you take into your body. If your immunity was weak, you'll get cancer even you eat vegetables,*" One 70-year-old-man discussed about the cause of cancer. A 62-year-old man from newly-infected group also supported that; "*Some people have a very healthy lifestyle. Sadly, they died at 40 or 50 years from cancer. It doesn't make any sense and it's proven that they're related*"

The villagers acknowledged the transmission process of the parasite. They knew that once stool contaminated with parasite eggs were released in a natural water source, the eggs hatched and continued its life cycle.

Some of them mentioned that public excretion could be avoided and in fact most of them used toilets on a regular basis, but some factors could not prevent disease transmission.

"*I saw the toilet pumping service car dumped stool in the river,*" one 70-year-old man mentioned about how the authorities managed the public toilet pumping service.

4. Perception of OV infection and its consequences

Table 4 Perception of OV infection and its consequences

Themes	Concerning topics	Emerging issues
• Cooked food was considered hygienic	• The infection was easy to threat	• Stigmatization from being infected
• Doctors were responsible for peoples' health	• Primary prevention was not a primary concern	• School was an information hub

The villagers perceived that OV infection was the major problem to the community (Table 4). They showed better attitudes towards OV infection. Although some of them might have misunderstood about the life cycle, they agreed that cooked food was more hygienic and could prevent not only OV infection but other intestinal parasites as well. Their consumption behaviours might be difficult to observe in practice, but parents were trying not to let their children consume *Koi pla*; "*Cooked food was good, vegetables need to be washed as well*"

After receiving stool examination results, some villagers decreased their uncooked fish consumption as they mentioned, "*Though I've never seen the worms, the result makes me stop eating raw fish*". Even when the result was negative, some were still concerned about other's results or they were afraid that if they chose to maintain risk behaviours. One day the result might turn to be positive, "*I see doctors collect stool and give medicine; I think it's time to stop eating raw fish. I don't want to be one of the infected*"

Some were concerned about possible side effects of taking praziquantel. They were afraid that if they were infected, they would have to take the medication again, "*It didn't feel good after taking the medicine*," said one 48-year-old from the re-infected group.

The younger generation had better accessibility to information. The school taught them about basic hygiene. Most households could not afford the internet service and IT infrastructure was still under development. However, internet was available at school and internet cafés in community centres. Some of them spent time during break or after class looking for more information about OV infection, "*I saw the pictures of worms on the internet*," said one school-age participant.

As the villagers perceived that OV infection was a disease, they preferred to focus on treatment rather than prevention. In addition, some of them learned that treatment for the infection was simple and feasible so they were willing to wait for the healthcare worker as they thought it should be the physician's responsibility to take care of them; "*Only the doctors know the disease, so it was better to leave it in their hands*" said one 70-year-old man.

5. Health concern of risk factors and the infection

Table 5 Health concern of risk factors and the infection

**Themes**	**Concerning topics**	**Emerging issues**
• Infection threats the community<br>• Each generations approach to disease threat in different ways	• Asymptomatic manifestation affects health awareness<br>• Health concerns do not associate with eating habit	• The term "liver fluke" was a misnomer since the liver was not directly involved<br>• Concern was primarily raised by the doctors

Table 5 reveals the health concerns of risk factors and the infection, high prevalence and incidence of OV infection triggered concerns about the health consequences to the community. However, they suggested that the main clinical manifestation was asymptomatic and presented symptoms were also nonspecific. They understood that OV infection caused abdominal distension, ascites and jaundice and were actually the symptoms of cholangiocarcinoma or liver diseases. They also knew that OV infection could be cured by anthelminthic medication. Most of them acknowledged the health threat of the infection. Because they were physically healthy, health awareness seemed to decrease due to the disease's natural history.

All groups expressed that they were asked for stool examination because the doctors (research team) thought it was important. Practically, nobody ever asked for a stool examination at the local healthcare facility, "*I consider what the doctor concerns*"

However, some thought to look after the health was the doctor's responsibility.

"*I'll do whatever they say. They said I have the parasite, so they gave me a drug.*" said one 70-year-old man.

Most villagers were not sure about the relationship between OV infection and cholangiocarcinoma. It was noticeable that the villagers also expressed various opinions toward the parasite and infection with respect to their age; teenage, adult or elderly.

Teenagers and adults knew that chronic infection could lead to cancer, but they did not know exactly what kind of cancer resulted from OV infection. Moreover, the name "liver fluke" (also called in Thai) confused the villagers and that the pathology occurred in liver tissue. Some believed that consuming *Koi pla* with alcohol could accelerate the process of liver cirrhosis. Elderly members thought OV infection was not related to cholangiocarcinoma. They observed that many were still healthy even though they regularly consumed uncooked fish.

Although all age groups realized the medical importance of OV infection, younger aged members were concerned about live worms living in their body while they were less aware of being sick from the infection. Male youths started to consume uncooked fish in adolescence to imitate their friends. Small children might be fed uncooked fish by their parents.

Adults and the elderly paid more attention to infection from the parasite. Lastly, the elderly members were concerned about both parasite and the infection. However, some admitted that they could not avoid eating uncooked fish despite knowing the unhealthy consequences.

The villagers stated that uncooked fish consumption needed to be slowly reduced. Abruptly discontinuing consumption seemed impractical. For *Koi pla* consumption, quantity and frequency were decreased over time except in the elder group that stated it was difficult to give up their way of life. Moreover, some of them mentioned that contributing to a major change was not worthwhile because they only had a few years left in their life. "*I've been living so far and I all have to die anyway, why not just enjoy life while it lasts*," said one 83-year-old woman from the re-infected group.

6. Diagnosis and treatment

Table 6 Diagnosis and treatment

Themes	Concerning topics	Emerging issues
• OV infection was curable	• Praziquantel was not available in the local health facilities<br>• Misunderstanding of anthelmintic drug	• Treatment causes more symptoms than infection

From Table 6, standard treatment with praziquantel was provided by the research team under the project after the stool was examined. OV infection cases received their diagnosis with praziquantel and other parasitic infections also received corresponding medication.

When they were aware of being infected, two approaches allowed them to receive medication. Firstly, they waited for the stool examination result from the research team who regularly visited them. They would definitely receive praziquantel with this method, "*Doctors come very often. If I got infected, then they'll give me drugs,*" said one 47-year-old woman in the local volunteer group.

Secondly, they also perceived that anthelminthic medication was available at the community healthcare centre or they could directly purchase over-the-counter medicine from the pharmacy for their convenience. Unfortunately, those anthelminthic drugs were for intestinal helminths such as albendazole or mebendazole. Praziquantel was not available in the community pharmacies unless villagers went to the district hospital which was 1 hour away.

Additionally, some thought that a single drug could cure all parasitic infections including OV infection. Especially for those who currently consumed uncooked fish, some purchased medicine from a drugstore and misunderstood that it could cure liver flukes so they returned to resume their eating behaviours, "*It's simple, just go to the drug store*," Said one 61-year-old from the newly-infected group. After receiving praziquantel, some felt that the infection was truly eradicated from their body, "*After I took your drug, all worms were killed. So I feel I'm stronger,*" said one 60-year-old woman from the previously-infected group.

However, some who used to take medicine still recognized praziquantel from its side effects, "*The drug was so strong, I vomited until nothing was left in my stomach*," said one 46-year-old from the local volunteer group.

Some stated that a home remedy was available to cure parasitic infection, "*From the old days, I mixed salt with coconut cream to treat parasite*s"

7. Role of National Control Program

Table 7 Role of National Control Program

Themes	Concerning topics	Emerging issues
• National control program generally improves heath<br>• Only fresh-prepared uncooked fish can cause the infection	• Avoidance of all uncooked fish menus was not practical	• Primary prevention needs more specific strategy

As shown in Table 7, the participants stated that they had better knowledge after they received health education. Consumption of *Koi pla* decreased, even some could not definitely quit but could reduce in terms of frequency and amount of consumption.

"*The doctors come and talk about it every year. I see the health campaign posters every day, it keeps reminding me not to eat Koi pla,*" said one 54-year-old from the previously-infected group.

Because the development of cholangiocarcinoma is a long process and mainly asymptomatic, so the cancer was not a major concern to the villagers after being infected with OV infection. In fact, the prevention was simple. Avoidance of uncooked fish did not require sophisticated medical intervention. Moreover, the treatment was also effective and affordable. At this point, some of them admitted that they did not pay much attention to cholangiocarcinoma, "*I can seek healthcare whenever I think I'm getting the infection*," said one member of the adult group.

The National Control Program has focused on the control of uncooked fish consumption, which would interrupt the infection process; therefore, it could potentially prevent the occurrence of cholangiocarcinoma from OV infection. However, the consumption behaviours were strongly attached to

the local culture, "*There were many raw fish dishes, I can't avoid all of them*," some mentioned about uncooked fish dishes. The control program suggested that all uncooked fish dishes should be avoided.

The group discussion agreed that when a particular dish was identified as a risk factor, it would be easier and more promising for behavioural modification.

Children had a better understanding about the knowledge of infection. Some adults and elderly felt more comfortable and hesitated less to be educated from their offspring. Children served as an effective medium for transferring knowledge. Additionally, they did not want their children to practice the same habit.

"*It was a good strategy. They're ashamed if they do not behave as role models,*" said one 38-year-old female from the adult group.

Most villagers had toilets in their house. Some of them installed toilets on their farm. Otherwise they would excrete in the field and cover with soil. Just a few people excreted directly in the natural water resource.

"*Toilets are everywhere, nowadays. Those who can't find one use a spade to cover it with soil*," one 55-year-old local health volunteer mentioned about how to excrete in the paddy field without toilet. "*I would hold it until I get home*," said one 18-year-old from the never-infected group.
